# Functional remapping in networks of the Parkinsonian brain: A preclinical neuroimaging perspective with clinical correlates

**DOI:** 10.1515/tnsci-2025-0374

**Published:** 2025-06-14

**Authors:** Zhuo Wang, Michael W. Jakowec, Giselle M. Petzinger, Daniel P. Holschneider

**Affiliations:** Department of Psychiatry and Behavioral Sciences, University of Southern California, 1975 Zonal Avenue, KAM 400, MC9037, Los Angeles, CA 90089-9037, United States of America; Graduate Program in Neurosciences, University of Southern California, Los Angeles, California, United States of America; Mann School of Pharmacy and Pharmaceutical Sciences, University of Southern California, Los Angeles, California, United States of America; Department of Biomedical Engineering, University of Southern California, Los Angeles, California, United States of America; Department of Neurology, University of Southern California, Los Angeles, California, United States of America

**Keywords:** Parkinson’s disease, exercise, functional brain mapping, plasticity, neurorehabilitation

## Abstract

Parkinson’s disease (PD) is increasingly understood as a neurodegenerative condition whose pathology extends beyond the direct and indirect basal ganglia pathways. Clinically, patients are all too painfully aware of dysfunction not only of motor circuits but also of somatosensory, autonomic, cognitive, and emotional systems. Functional neuroimaging studies have begun to document a functional reorganization in the PD brain across a wide number of networks. In particular, the cerebellar-thalamocortical, as well as the fronto-striatal circuit, have been shown to undergo functional reorganization. In this narrative review, citing preclinical as well as clinical neuroimaging studies, our objective is to highlight trends and discuss the relevance of cerebral adaptive changes. It remains clear that not all changes contribute to the normalization of functions. Parsing differences between functional “compensation,” “silencing,” or “maladaptation” in neural circuits is important. A necessary next step in neurorehabilitation is the question of whether compensatory cerebral changes can be enhanced. In this regard, physical exercise remains of interest, given that in patients, exercise may allow some degree of symptom improvement and possibly slow the course of the disease. Future interventions may wish to integrate neuroimaging findings as potential targets to support neuroplastic changes.

## 1 Introduction

Originally described in the scientific literature on cerebrovascular stroke under the term “diaschisis,” it is well known that focal brain damage can extend beyond the direct site of injury to affect remote brain networks [1]. In its original conception, diaschisis was the result of downstream pathologic changes seen at second-order neurons in the cerebral network due to the dying back of efferent fibers, as well as afferent fiber remodeling. Depending on lesion size and location, changes in cerebral functional activation might or might not be apparent in the subject’s quiescent state, often becoming unmasked or amplified only during an acute behavioral or physiologic challenge. The neuroadaptive conceptual framework underlying diaschisis is also relevant in Parkinson’s disease (PD). In this translational narrative review we examine principles of cerebral functional reorganization in the rodent model of Parkinsonism, drawing parallels between the preclinical model and clinical symptoms, as well as neuroimaging findings in animals and in human PD subjects. Our objective is to highlight trends and discuss the relevance of cerebral adaptive changes. Given the fact that the preponderance of clinical neuroimaging studies in PD focus on the motor system, our discussion of adaptive change in the preclinical model focuses on the 6-hydroxydopamine rodent model, which parallels motor deficits remarkably well [2,3]. We discuss how cerebral reorganization may be adaptive, maladaptive, or include functional silencing. A “next step” in the neurorehabilitation of PD patients is the question of whether compensatory cerebral changes can be enhanced and whether physical exercise or other neuroadaptive treatment strategies might be engaged to provide a more targeted intervention.

## 2 Neuroadaptation following dopaminergic deafferentation

**Figure 1:**
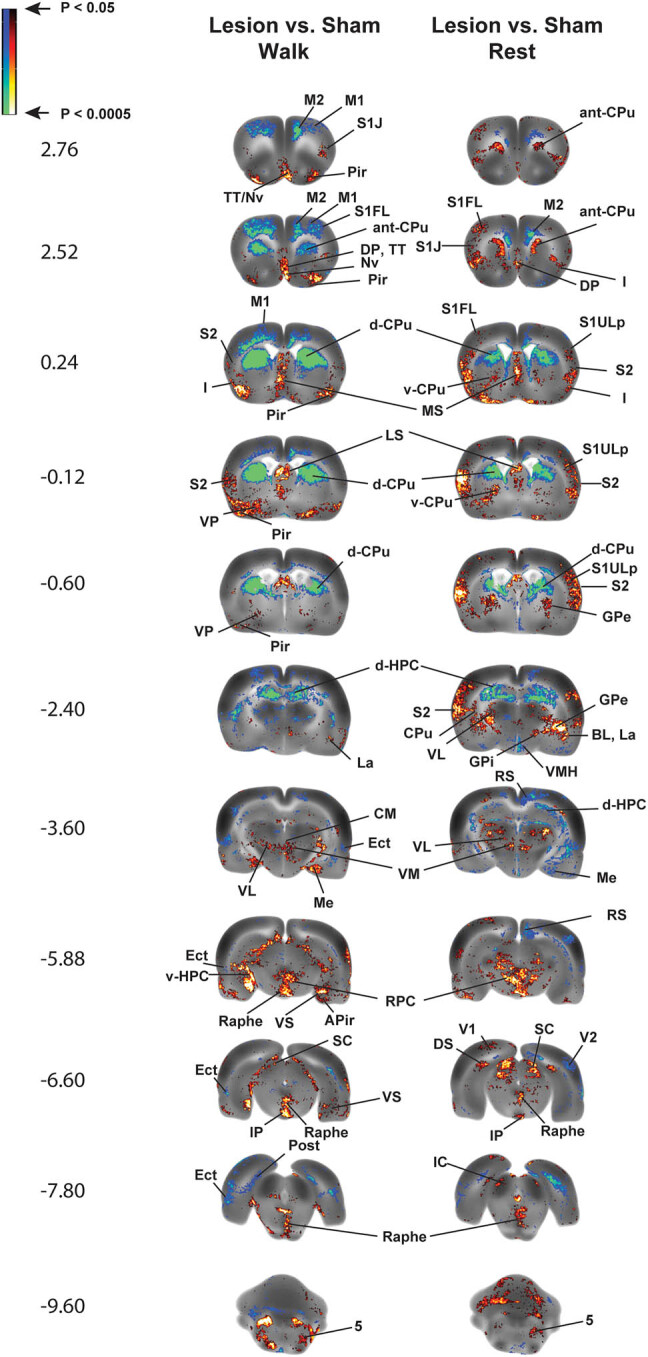
Regions of statistically significant differences of functional brain activation in rats with bilateral 6-hydroxydopamine striatal lesions compared to sham-lesioned rats. Statistically significant lesion effects on regional cerebral blood flow (rCBF) during acute treadmill walking (Lesion/Walk, *n* = 9 vs Sham/Walk, *n* = 10) or at rest (Lesion/Rest, *n* = 10 vs Sham/Rest, *n* = 9) in rats are shown. The comparison highlights lesion effects (Lesion vs Sham). A selection of representative coronal slices (anterior–posterior coordinates relative to bregma) is shown. Colored overlays show statistically significant positive (red) and negative (blue) differences in rCBF. Abbreviations are those from the Paxinos and Watson rat atlas [5]: 5 (trigeminal n., motor, sensory), aca (anterior commissures), BL (basolateral amygdalar n.), Ce (central amygdalar n.), Cg (cingulate cortex), CM (central medial thalamic n.), CPu (CPu: anterior, ant-CPu; dorsal, d-CPu; ventral, v-CPu), d-HPC (dorsal hippocampus), DP (dorsal peduncular cortex), DS (dorsal subiculum), Ect (ectorhinal cortex), Ent (entorhinal cortex), GPe (external globus pallidus), GPi (internal globus pallidus/entopeduncular n.), I (insular cortex), IC (inferior colliculus), IL (infralimbic cortex), IP (interpeduncular n.), La (lateral amygdalar n.), LO (lateral orbital cortex), LP (lateral posterior thalamic n.), LS (lateral septum), M1, M2 (primary, secondary motor cortex), Me (medial amygdalar n.), MS (medial septum), mRT (mesencephalic reticular formation), Nv (navicular n.), PaS (parasubiculum), PH (posterior hypothalamus), Pir (piriform cortex), Pn (pons), PrL (prelimbic cortex), PtA (parietal association cortex), RPC (red n.), RS (retrosplenial cortex), S1DZ, S1FL, S1J, S1Tr, S1ULp, (primary somatosensory cortex: dysgranular, forelimb, jaw, trunk, upper lip), S2 (secondary somatosensory cortex), SC (superior colliculus), SN (substantia nigra), STN (subthalamic n.), TT (tenia tecta), vermis (2nd, 3rd cerebellar simple lobule), V1, V2 (primary, secondary visual cortex), v-HPC (ventral hippocampus), VL (ventral lateral thalamic n.), VM (ventromedial thalamic n.), VMH (ventromedial hypothalamus), VPL/VPM (ventral posterolateral, ventral posteromedial thalamic nuclei), VP (ventral pallidum), VS (ventral subiculum), ZI (zona inserta). Figure adapted from Wang et al. [6] under Creative Commons Attribution 4.0 International License (http://creativecommons.org/licenses/by/4.0/) with permission from the authors.

Despite their differences, remarkable similarities exist between normal rodents and humans at the level of brain anatomy and motor processing, with an overall conservation of supraspinal networks [4]. In the preclinical model of Parkinsonism that follows partial bilateral dopaminergic deafferentation of the dorsolateral striatum (caudate-putamen, CPu), regional changes in cerebral blood flow (rCBF) of the CPu when assessed at rest may show only small decreases in secondary motor cortex (M2) and at the dorsal CPu lesion site, with CPu anterior to the lesion actually showing small increases in rCBF (Figure 1, rows 1–2). However, when imaged during a motor challenge (walking), decreases in rCBF are noted broadly across the basal ganglia-thalamocortical (BG-T-C) circuit, including the anterior medial CPu, the ventrolateral motor thalamus, as well as the primary and secondary motor cortices (Figure 1, rows 1–6) [6]. It is surmised that deficits in the motor circuit become accentuated during motor challenges due to the inability of regional perfusion to accommodate increases in local metabolic demand associated with increases in neuronal firing. Similar observations have been made in PD patients performing a motor task [7,8] or during a dual-task challenge in which the patient typically is required to simultaneously perform a motor task (e.g., foot or finger tapping) and a cognitive task (e.g., counting backward by 3 s), something often used to assess motor control and cognitive–motor interference [9].

Concomitant with such pathologic deficits, neuroimaging studies have revealed novel functional brain activations in the PD brain, both at rest and during the performance of a task. It has been proposed that in response to local functional deficits, such adaptive strategies represent an effort by the brain to “recruit” additional cerebral regions [10]. Such “compensatory” strategies in the PD brain to reduce motor deficits may include (i) increased reliance on residual, nonlesioned neurons in the affected motor circuits; (ii) transfer of function to other types of neurons in the affected motor circuits; (iii) increased reliance on contralateral circuits (in cases of asymmetric disease); and (iv) learning of alternate behavioral strategies for executing a given task, with transfer of function to alternate circuits.

In the preclinical model of partial bilateral dopaminergic deafferentation of the dorsolateral CPu, lesions compared to sham animals show such neuroadaptive changes. These include the following:

- *An increased engagement of nonlesioned neurons in the affected BG-T-C circuit:* Here, a relative increase in rCBF in response to neural activity is seen in the globus pallidus (rest, walking) (Figure 1, rows 5 and 6), the motor nuclei of the thalamus (ventromedial, central medial) (Figure 1, row 7), and to a lesser extent in the zona incerta, subthalamic nucleus, and substantia nigra (rest).
- *A functional reorganization of the CPu:* In the animal model, alongside an expected loss of functional connectivity between the dorsolateral CPu and secondary motor cortex, we see newly established functional correlations between regions of primary and secondary motor cortices with the anterior and ventrolateral CPu, both regions outside the dorsolateral striatal lesion site [11]. A functional reorganization of the CPu has been previously reported also in PD subjects but not in controls during a single task (e.g., motor sequence task, eye movement) [12,13] or dual-task performance (e.g., ankle movement + cognitive task) [14], as well as in the resting state [15–18].
- *Functional recruitment of associated motor regions, including the superior colliculus and red nucleus (rest, walking)* (Figure 1, rows 8 and 9): The superior colliculus plays a central role in the suppression of the eyeblink reflex. Inhibitory modulation of the superior colliculus by the substantia nigra has been suggested to explain decreases in the spontaneous blink rate and a hyperexcitable stimulus-triggered blink reflex in the 6-hydroxydopamine (6-OHDA, a dopaminergic neurotoxin) rodent model [19,20], as well as in human PD subjects [21–23], with alterations in functional activation in both [6,24]. In the red nucleus, a structure in the rostral midbrain involved in motor coordination, a previous study has shown an increase in glucose utilization in striatally lesioned rats [25,26], with alterations in structure and function also noted in PD subjects [27,28].
- *Functional reorganization of somatosensory cortex, demonstrating both increased and decreased rCBF in subregions of primary and secondary somatosensory cortices at rest and during walking* (Figure 1, rows 4 and 5): Such a “diffusion” or remapping of cortical sensorimotor activity suggests that somatosensory cortex may have to “work harder” after striatal damage in order to provide adequate sensory processing to the animal, both at rest and during treadmill walking. Consistent with this cortical remapping is an increase in the rCBF of the ventral posterolateral nucleus of the thalamus, a primary thalamic relay for somatic sensory, i.e., tactile/kinesthetic and nociceptive information from the trunk and limbs [29], which provides direct projections to the caudal CPu [30]. Increases are noted bilaterally, both at rest and during the locomotor challenge, and may indicate increased neural activity whose aim is to boost neural sensory input to a hypofunctional CPu. Indeed, others have reported somatosensory abnormalities in the 6-OHDA rat model [31], as well as somatosensory deficits in patients with PD [32–34].
- Functional reorganization of the cerebellar-thalamocortical pathway is discussed below.

In addition to processing motor information, an important interaction exists between the basal ganglia and the limbic system [35]. In our rodent model, dopaminergic deafferentation results in a significant increase in rCBF in select regions in the mesolimbic circuit, including broad areas of the amygdala, ventral hippocampus, ventral CPu, nucleus accumbens, and tegmental nucleus. Also noted is a significant increase in rCBF in lesioned compared to sham-lesioned rats in the raphe, septum, insula, piriform, and entorhinal cortex, and the dorsal endopiriform nucleus (Figure 1, rows 4, 6–10). Dysregulation of functional activation of limbic structures has been previously reported in PD subjects [36–38] and may be related to the strong association of depression with PD [39,40].

The above findings support a recognition, originally formulated by Jellinger [41] but long appreciated by patients themselves, that PD is a multisystem disorder involving a broad range of clinical symptoms across motor, sensory, postural, muscular, gastrointestinal, emotional, and cognitive domains [42–44]. This suggests that though the basic features of the “classic model” based on the direct and indirect pathways of the basal ganglia-thalamocortical (BG-T-C) circuit have stood the test of time, it does not adequately integrate neural adaptive strategies available to the brain. A re-evaluation of the functional anatomy of the circuits in the Parkinsonian state is needed.

### 2.1 Importance of the cerebellar-thalamic-cortical circuit after basal ganglia injury

Though much attention has been given to the basal ganglia-thalamocortical (BG-T-C) circuit in PD, clinical observation, as well as preclinical models, suggests that following dopaminergic deafferentation of the CPu, there is a functional reorganization in the cerebellar-thalamocortical circuit (Cb-T-C). Increased reliance in PD on the Cb-T-C circuit is underscored by fascinating clinical phenomena in PD, which is that motor deficits can be partially overcome when motor activity is externally guided by visual or auditory cues [45–48]. The distinction between internally and externally guided movement has been proposed to relate to the differential recruitment, respectively, of the BG-T-C and Cb-T-C circuits [49,50]. Based on the results of fMRI studies, Lewis et al. [8] and others [51–54] have highlighted the dominance of the Cb-T-C circuit during the performance of externally guided motor movement (e.g., finger tapping guided by an external metronome), and contrasted this with the dominance of the BG-T-C circuit during the performance of internally guided motor movements (e.g., finger tapping performed as internally prerehearsed sequences).

**Figure 2:**
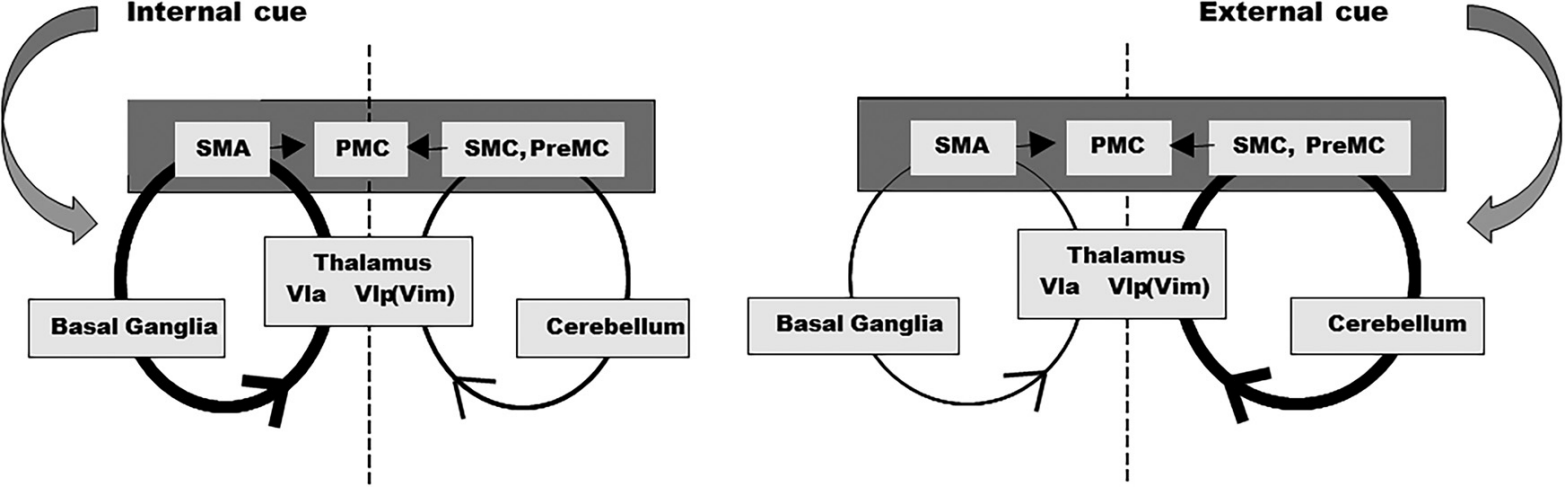
Basal ganglia-thalamic-cortical and cerebellar-thalamic-cortical circuits during externally or internally cued tasks. PreMC, lat. premotor cortex; PMC primary motor cortex; SMA, supplementary motor area; SMC, somato-sensory cortex; Vla ventrolateral anterior thalamic n.; Vlp (Vim) ventrolateral posterior thalamic n. (ventralis intermedius). A difference between externally or internally guided tasks is often related to the difference between internal and external timekeeping systems. Thickness of the line denotes dominance of the pathway. Adapted from Lewis et al. [8] with permission from Elsevier.

It has been proposed that BG-T-C and Cb-T-C circuits are in balance (Figure 2). Under normal conditions, externally guided tasks are primarily processed through Cb-T-C circuitry, with secondary recruitment of the BG-T-C circuitry, whereas internally guided tasks are primarily encoded in the BG-T-C pathway, with secondary recruitment of Cb-T-C circuitry [51]. The motor deficits of PD are related primarily to the volitional initiation of movement that is required for the performance of internally guided tasks. Internally guided tasks in PD show reduced activation of the BG-T-C pathway (its primary processing center) [8,55]. It has been proposed that increased recruitment of the Cb-T-C path during internally guided tasks allows for partial compensation of deficits in the BG-T-C path at the cortical level [56–58]. While it is not disputed that the BG-T-C and Cb-T-C pathways represent anatomically segregated loops that communicate via the thalamus and converge at the level of the cortex, the question of interdependence or independence remains an active research question [59,60].

A significant number of publications, mostly from the clinical neurosurgical and neuroimaging literature, have supported the idea that PD patients off medication may compensate for their basal ganglia-cortical loop dysfunction using motor pathways involving the cerebellum [57,61–70]. In fact, the past 5 years (2020–2025) have seen 70 publications with “Parkinson’s” and “cerebellar or cerebellum” words in titles, with 15 of these focused on external cerebellar stimulation as a treatment intervention. Functional imaging studies examining PD subjects at rest have noted increased perfusion in the cerebellum, the thalamus, and putamen/globus pallidus, with decreased perfusion noted in the premotor and posterior parietal cortex [66,71]. These observations are also supported by functional connectivity studies, which have shown significantly increased functional connectivity in PD compared to normal subjects in the left cerebellum, left primary motor cortex, and left parietal cortex and decreased functional connectivity in the supplementary motor area, left dorsal lateral prefrontal cortex and left putamen [67]. When PD subjects undergo a motor challenge (usually finger tapping or a reaching task), the hyperactivation of the cerebellum, thalamus, and primary motor cortex is accentuated relative to controls [57,63,64,72–76]. There is increased recruitment of the Cb-T-C circuit concomitant with PD progression. Cerebellar hyperactivation increases monotonically with an increased movement speed of the motor challenge [58,64] and shows partial normalization alongside the improvement of motor deficits following levodopa administration [68,77]. Such correlation studies suggest that hyperactivation of the cerebellum and motor cortex represents a compensatory mechanism for hypoactivation in the basal ganglia rather than a nonspecific pathophysiological sequelae of the disease [69]. These findings suggest the possibility that cerebellar projections to the BG-T-C circuit could potentially boost its activity. This might occur through disynaptic cerebellar projections to the thalamus, or through monosynaptic projections to the midbrain dopaminergic nuclei, the ventral tegmental area, and the substantia nigra pars compacta [78–82]. Our studies in the preclinical model of Parkinsonism confirm an increased reliance on the Cb-T-C circuit. Rats with bilateral dorsolateral CPu lesions compared to normal rats when imaged at rest show increased rCBF in the vermis, deep cerebellar nuclei, and ventrolateral thalamic nucleus, with a decrease in this effect during motor challenge [6].

## 3 Effect of physical exercise on neural circuits

### 3.1 Exercise-related functional changes in the normal brain

#### 3.1.1 Exercise alters cerebral function

A range of molecular and cellular mechanisms by which exercise modifies brain function have been amply reported. These include increased glial cell volume, angiogenesis, changes in neurotransmitter levels, changes in neurotransmitter receptor density, the expression of endogenous neurotrophins, the growth of neural processes, and neurogenesis [83,84]. While changes may be observed widely across the brain, research suggests that exercise effects are accentuated in specific brain regions that represent nodes in wider networks. Neuroplastic molecular changes have been documented to occur within the motor circuit, with a large literature also showing functional changes in the hippocampus and cerebellum. It is no surprise that this dramatic remodeling is reflected in changes in functional brain activation, as revealed by neuroimaging.

#### 3.1.2 Chronic exercise conceptualized as a neuroadaptive change to repeated acute motor challenge

Of note, changes in functional brain activation observed after chronic exercise are frequently opposite to those elicited during acute motor challenge, where previous work has shown increases in rCBF [85] and regional cerebral glucose metabolism (rCMR) [86] in motor regions (motor cortex, CPu, substantia nigra, and cerebellum), and decreases in rCMR in limbic regions (including amygdala, hippocampus, hypothalamus, and dorsal raphe). This is relevant insofar as the term “exercise” appears awkwardly in the literature, both in relationship to an acute motor challenge and to chronic motor training. Though significant performance improvements can be seen after only brief exposure to exercise, the changes in cortical reorganization typically require days to weeks and occur only after a behavioral asymptote has been achieved and improvements in motor function have reached a plateau [87]. Given the different applications of the term “exercise,” it is understandable that activation patterns associated with practice and repetition of motor movements in human subjects remain incompletely understood, with both increases, no change, and decreases reported. Factors influencing this heterogeneity include not only the timing of the imaging in relation to the duration of training but also the type of exercise, variability in the duration and intensity of exercise exposure, and the nature of the activation procedure during which brain imaging is performed.

#### 3.1.3 Exercise and extensive motor training enhance the metabolic efficiency of the motor circuit

**Figure 3:**
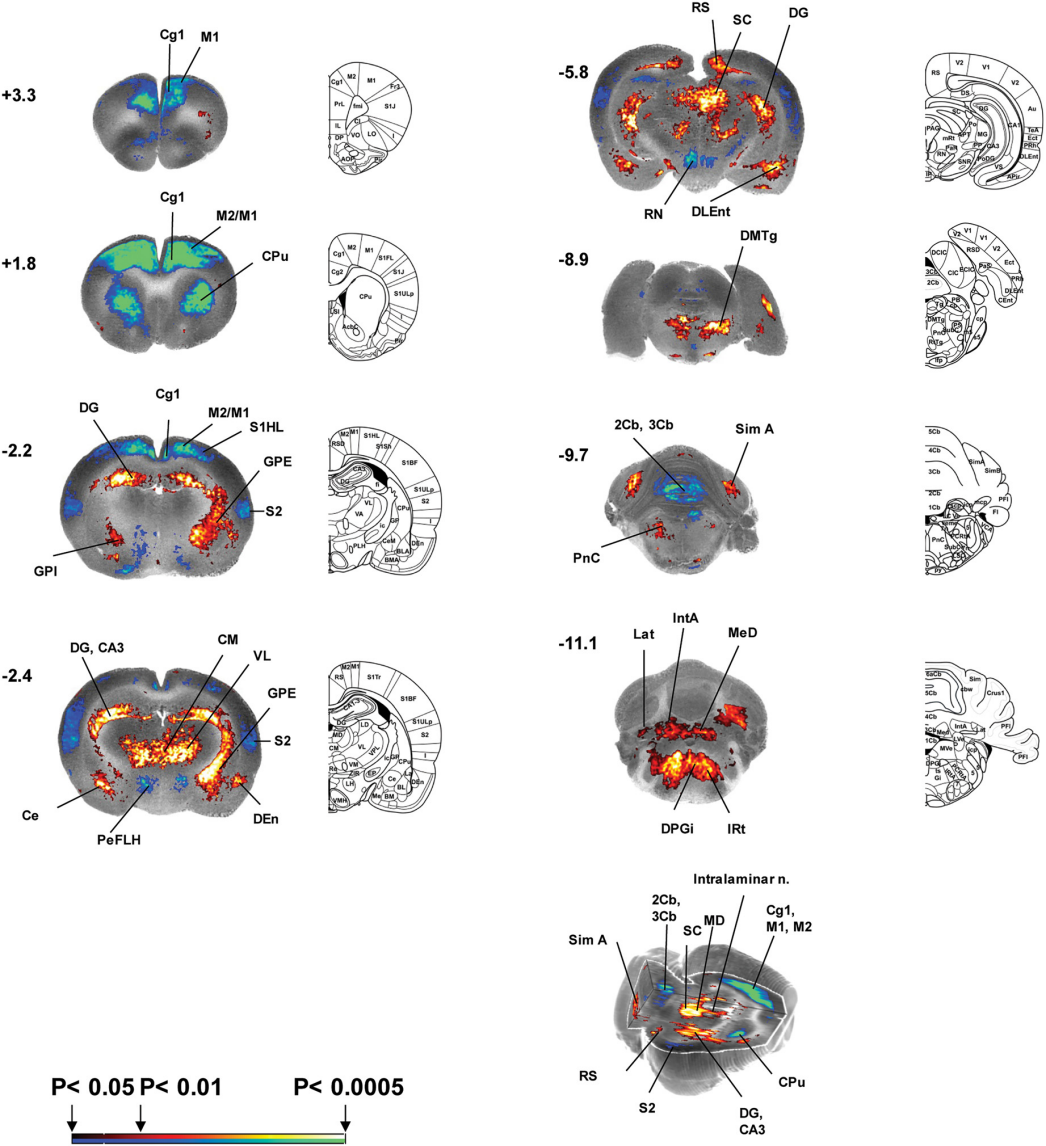
Regions of statistically significant differences of functional brain activation during treadmill walking in previously exercised (*n* = 10) and nonexercised (*n* = 10) normal rats. A selection of representative coronal slices (anterior–posterior coordinates relative to bregma) is shown. Colored overlays show statistically significant positive (red) and negative (blue) differences in cerebral blood flow (voxel level, *p* < 0.05, clusters < 100 contiguous voxels). Line drawings have been adapted from the Paxinos and Watson (2005) rat atlas with permission from Elsevier: Exercise training results in (i) decreased activation in M1, M2, CPu, and midline cerebellum and (ii) increased activation in globus pallidus, thalamus (VL, CM), and subthalamic n. Abbreviations: 2Cb, 3Cb (2nd and 3rd cerebellar lobules), Au (auditory cortex), CA3 (hippocampus CA3 region), Ce (central nucleus of the amygdala), Cg1 (cingulate cortex area 1), cp (cerebral peduncle), CPu (CPu), DEn (dorsal endopirform nucleus), DG (dorsal geniculate of the hippocampus), DLEnt (dorsolateral entorhinal cortex), DMTg (dorsomedial tegmental area), DPGi (dorsal paragigantocellular nucleus), GPE (external globus pallidus), GPI (internal globus pallidus), Hb (Habenula), IRT (intermediate reticular nucleus), IntA (interposed cerebellar nucleus), Lat (lateral or dentate cerebellar nucleus), M1, M2 (primary, secondary motor cortex), MD (medial thalamic nucleus), MeD (medial or fastigial cerebellar nucleus), PeFLH (perifornical part of the lateral hypothalamus), PF (parafascicular thalamus), PnC (pontine reticular nucleus, caudal), RN (red nucleus), RS (retrosplenial cortex), S1HL (primary somatosensory cortex, hindlimb), S2 (secondary somatosensory cortex), SC (superior colliculus), Sim A (simple lobule of the cerebellum), VL (ventrolateral thalamus), ZI (zona incerta). Figure adapted from Holschneider et al. [95] with permission from Elsevier.

There is now extensive evidence in animals that exercise training can induce both structural and functional adaptation (“plasticity”) within motor areas, including the motor cortex [50,88], basal ganglia [89–91], cerebellum [92,93], and red nucleus [94]. In our experiments on normal rodents, animals received 6 weeks of Rotarod exercise and were subsequently imaged during a locomotor challenge. Compared to nonexercised controls, exercised animals showed decreases in regional rCBF in the motor pathway (primary/secondary motor cortex, dorsolateral CPu, zona incerta, cerebellar vermis) and primary somatosensory cortex, as well as increases in limbic regions (hippocampus, entorhinal cortex, periaqueductal gray, and amygdala) (Figure 3) [95]. Similar results have also been reported in human subjects, with decreased activation in motor-related regions after long-term motor training [96–98].

Of greatest consistency in this literature are human studies comparing functional brain activation of trained musicians with novices during the performance of finger sequences (reviewed in the study of Munte et al. [99]). Here, the extensive skill training of the musicians results in an increased size of the hand motor cortex [100] and corpus callosum [101], reduced transcallosal inhibition [102], and structural adaptation of the cerebellum [103]. However, in professional musicians, the magnitude of the activational response to simple, overpracticed finger tasks is attenuated relative to that seen in novices [104–107]. This is true for cortex (motor, cingulate, and medial prefrontal), basal ganglia, the cerebellar vermis, and substantia nigra. Furthermore, in nonmusicians, attenuation of activation in somatosensory and motor cortices has been reported as subjects become more practiced on finger tasks [108]. When finger sequences are pre-learned, a lesser and more circumscribed activation has also been noted in the cerebellum (vermis and hemispheres) [109,110]. Similar effects on functional activation of the motor cortex, striatum, and cerebellum have also been made on training human subjects on skilled locomotor tasks [111]. These findings suggest that extensive motor training results in a functionally more efficient way to control movements [112]. The nature of this increase in functional efficacy needs further investigation but has been proposed to involve an increase in aerobic metabolic capacity through enhancement of the mitochondrial bioenergetics and biogenesis, including cytochrome oxidase [113–117], as well as a possible shift to increased lactate utilization [118,119].

**Figure 4:**
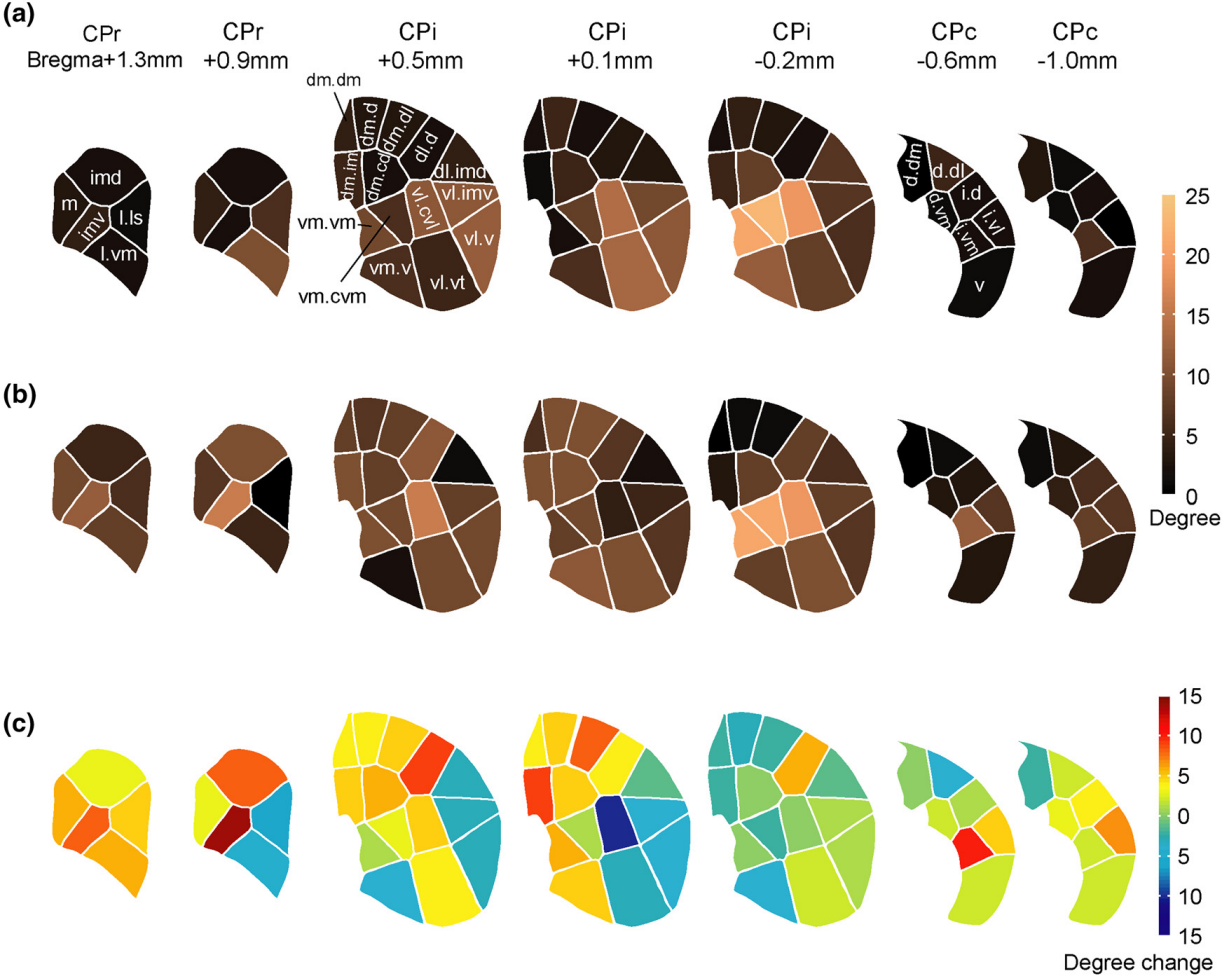
Connectivity degree changes in the *normal mouse* in subregions of the caudoputamen (CPu) in response to 6 weeks of horizontal treadmill exercise. Imaging was performed during walking on a motor-driven wheel. (a) The connectivity degree of CPu domains in the control (nonexercise) group is color-coded. Here, “degree” represents a measurement of the number of functional connections linking the node to other nodes in the cortico-basal ganglia-thalamic network. (b) Connectivity degree of CPu domains in the exercise group. (c) Connectivity degree changes in the exercise group compared to controls. Here, a negative number implies a higher connectivity number in the nonexercised mice across the cortico-basal ganglia-thalamic network. CPr/CPi/CPc, rostral/intermediate/caudal caudoputamen. Domain maps were drawn based on the mouse structural connectome [120]. Reproduced without change from Wang et al. [121] under Creative Commons Attribution 4.0 International License (http://creativecommons.org/licenses/by/4.0/) with permission from the authors.

The notion that exercise enhances the efficiency of the motor circuit receives further support from functional connectivity analyses. We examined the effects of 6 weeks of exercise on functional brain metabolic connectivity in the CPu of normal animals. We examined connectivity density for a structure, defined as the number of functional connections expressed as a percentage of the total number of possible connections (degree centrality) across the cortico-basal ganglia-thalamic network. As shown in Figure 4, exercise in normal mice elicited broad increases in intra- and inter-structural connectivity across the CPu. Noteworthy here are exercise-associated changes in connectivity density at the subregional level. In the exercise compared to the control group, the rostral level and dorsolateral aspect of intermediate-level CPu showed the greatest increase in functional connectivity. The rostral CPu exhibits high integration among cortical afferents across different cortical subnetworks, suggesting a role for exercise in cross-modality integration [120]. The dorsolateral aspects of the intermediate CPu receive input from cortical areas mapping the limbs [122], while dorsomedial CPu has prominent connections to the medial prefrontal cortex (anterior cingulate and prelimbic cortex) [123] and has been associated with set-shifting and reversal learning [124]. Changes in functional connectivity in these subregions of the CPu suggest a role for exercise in shaping limb function and cognition.

In addition to increased functional connectivity in the CPu, exercised normal animals compared to sedentary controls showed (i) increased positive connectivity within and between the motor cortex and caudoputamen (CP). These findings are consistent with reports in normal human subjects demonstrating increases in functional connectivity of the motor cortex following several minutes [125,126] or 4 weeks of motor training [127]. (ii) Newly emerged negative connectivity (i.e., an anti-correlation or inverse correlation between the activity of two brain regions) of the substantia nigra pars reticulata with the globus pallidus externus and CPu, and (iii) reduced functional connectivity of the prefrontal cortex (PFC). The findings of increased functional connectivity in the CPu and motor cortex in exercised normal animals in the context of decreases or no change in rCMR in these regions suggest that the motor regions, rather than being deactivated, function with greater integration and efficiency at the network level. Such dissociation between regional activity and functional connectivity has been previously reported. Eisenstein et al. [128] reported that in cognitively intact older adults, a physically active lifestyle was associated with higher memory performance and lower activity level of the hippocampus but higher functional connectivity of the hippocampus to hubs of the default mode network during memory encoding (hubs being brain regions with a significantly higher number of functional connections compared to other regions of the network). Conversely, Trujillo et al. [129] reported that nonexercised Parkinson’s patients, compared to healthy controls, showed increased activity but decreased functional connectivity in the dorsolateral prefrontal cortex during a visuospatial task. With these and other findings [130], one may hypothesize that a simultaneous task-related decrease in regional activity and increase in functional connectivity may be a marker for high functionality of the region, while an increase in activity coupled with a decrease in connectivity may represent a marker for dysfunction [121,131,132]. The idea of dissociation between regional activity and functional connectivity as a marker of regional brain function remains a hypothesis needing further testing, especially with regard to differential effects of factors, including the imaging conditions (resting state versus activational paradigms), as well as medications.

### 3.2 Exercise-related functional changes after dopaminergic deafferentation

**Figure 5:**
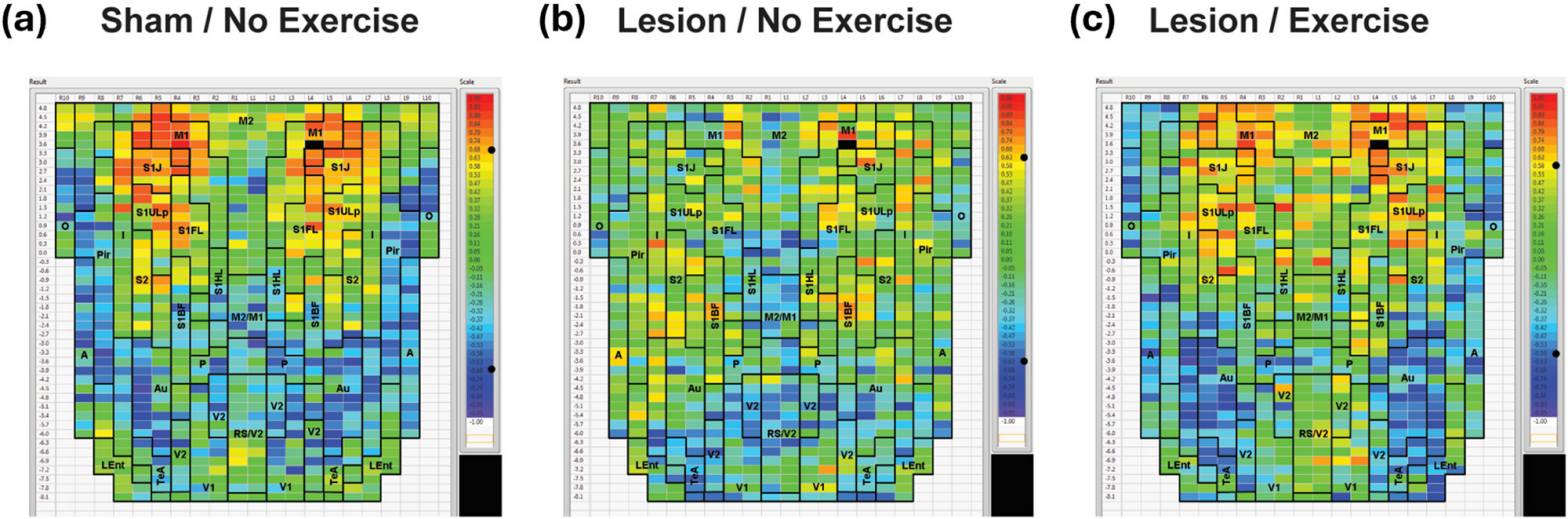
Exercise increases cortical functional connectivity of the primary motor cortex (M1) in the 6-OHDA lesion PD model. Rats received 6-OHDA lesions of the bilateral dorsolateral CPu, with controls being nonlesioned animals. Daily wheel running exercise was administered over 6 weeks, beginning 2 weeks postlesion. Thereafter, animals received intravenous injections of the cerebral perfusion tracer [^14^C]-iodoantipyrine during slow walking on a motorized horizontal treadmill. Following euthanasia, brains were cryosectioned and processed by autoradiography. Selection in each animal of 806 regions of interest (ROIs) along the cortical mantle across 34 coronal slices was used to generate cortical flatmaps. Color-coded maps of group averages in the flattened cerebral cortical surface are shown for the following groups (a) Sham/no exercise, (b) Lesion/No-Exercise, and (c) Lesion/Exercise. The rows denote coronal sections from anterior to posterior, with ROIs represented by cells and numbered starting from the midline. Right (R) and left (L) hemispheric ROIs are shown on the left and right sides of the figure, respectively. A functional seed is placed in the left anterior primary motor cortical area (M1) at bregma AP + 3.6 mm (black cell on the right side of each map). Each ROI is represented by a cell with its Pearson’s correlation coefficient with the M1 seed color-coded. Significant positive and negative correlations are denoted by red and blue colors, respectively. The critical value of correlation coefficient (*R*) for statistical significance (*p* < 0.05) is denoted by a dot (•) placed on the *R*-value color scale bar on the right. Abbreviations [141]: A, amygdala; Au, auditory; Fr3, frontal cortex area 3; I, insular; LEnt, lateral entorhinal; M1, primary motor; M2, secondary motor; O, olfactory; P, parietal; Pir, piriform; RS, retrosplenial; S1BF, primary somatosensory for the barrel fields; S1FL, forelimbs; S1HL, hindlimbs; S1J, jaw; S1ULp, upper lip region; S2, secondary somatosensory; TeA, temporal association; V1, primary visual; V2, secondary visual. Unlabeled regions represent transitional areas between two regions. Figure adapted from Peng et al. [140] under Creative Commons Attribution 4.0 International License (http://creativecommons.org/licenses/by/4.0/) with permission from the authors.

In the past 12 months, over two dozen meta-analyses have been published on the effects of chronic exercise on functional outcomes in PD. Though specific outcome measures vary, a shared conclusion of previous studies appears that exercise is a useful adjunct to the treatment of PD as it relates to specific gait parameters, quality of life, cognition, the regulation of mood, as well as possibly the dose requirements of l-dopa [133–139]. In the 6-OHDA animal model of striatal injury, 6 weeks of exercise exerts many of the neuroadaptive changes seen in the normal brain. Primary targets include the motor system, hippocampus, and limbic structures [6]. In the cerebral cortex in the rodent model of dopaminergic deafferentation of the dorsolateral CPu, exercise can substantially reverse the loss of functional connectivity density (degree centrality) in primary and secondary motors (M1, M2) and somatosensory (S1, S2) cortices, with little effect in nonsensorimotor regions such as visual, auditory, and piriform cortices [140] (Figure 5). In addition, long-term exercise in preclinical PD models elicits (i) partial normalization of many of the lesion-induced alterations in resting state functional connectivity, including reintegration of the dorsolateral CPu into the motor network; (ii) emergence of the ventrolateral CPu as a new broadly connected network hub; and (iii) increased resting state functional connectivity among the motor cortex, motor thalamus, basal ganglia, and cerebellum [11].

There are relatively few published brain imaging studies on the effects of long-term exercise in human PD; in most of these, imaging is performed with patients on their standard Parkinsonian medication regimens. A recent review that included seven studies on the brain activation patterns of PD in response to a variety of broadly defined exercise and motor training modalities and with patients imaged either at rest or during task performance concluded that exercise for PD results in broadly enhanced activation in subregions within the cerebellum (especially, anteriorly), putamen, frontal lobe, parietal lobe, and occipital lobe [138]. Exercise improves thalamo-motor cortical connectivity [142], modifies functional connectivity in the pedunculopontine nucleus (PPN, a critical cerebral locomotion center) [143], and alters the responsivity of the ventral CPu, likely related to changes in the mesolimbic dopaminergic pathway, with increases in evoked dopamine release [144] and dopamine D2 receptor binding potential [145]. In a resting state study in PD subjects, prior exposure to aerobic exercise (×3/week over 6 months), but not stretching, led to increased functional connectivity of the anterior putamen with the sensorimotor cortex relative to the posterior putamen. Behaviorally, aerobic exercise also improved cognitive control. Furthermore, aerobic exercise increased functional connectivity in the right frontoparietal network, proportionally to fitness improvements, while also reducing global brain atrophy [146]. Another resting state study using arterial spin labeling reported that after 2 months of exercise training, there was an increase in cerebral blood flow in the left primary motor cortex (M1), left supplementary motor cortical area, and left cerebellar cortex. Resting-state fMRI showed increased functional connectivity of the bilateral globus pallidus to the right central operculum, right posterior cingulate gyrus, and left sensorimotor cortex. Seed-to-voxel analysis demonstrated a functional connectivity between M1 and the left superior frontal gyrus. The left lateral cerebellum (crus II) showed strengthened connections with the left pre-rolandic area, left post-rolandic area, and left supramarginal area [147]. An fMRI study performed during a motor task showed that PD subjects who had undergone 16 weeks of high-intensity bicycle training compared to nonexercised controls had greater activation of the right fusiform gyrus and functional connectivity between the cingulum and areas of the frontal cortex and between the cerebellar vermis and the thalamus and posterior temporal gyrus [148].

## 4 Neuroadaptation and the role of interventions to optimize neurorestoration

A necessary “next step” in the neurorehabilitation of PD patients is the question of whether compensatory cerebral changes can be enhanced. This might entail interventions, including exercise, pharmacologic or dietary interventions, regional brain stimulation, or targeted stem cell transplants.

### 4.1 Can exercise enhance compensatory activity?

**Figure 6:**
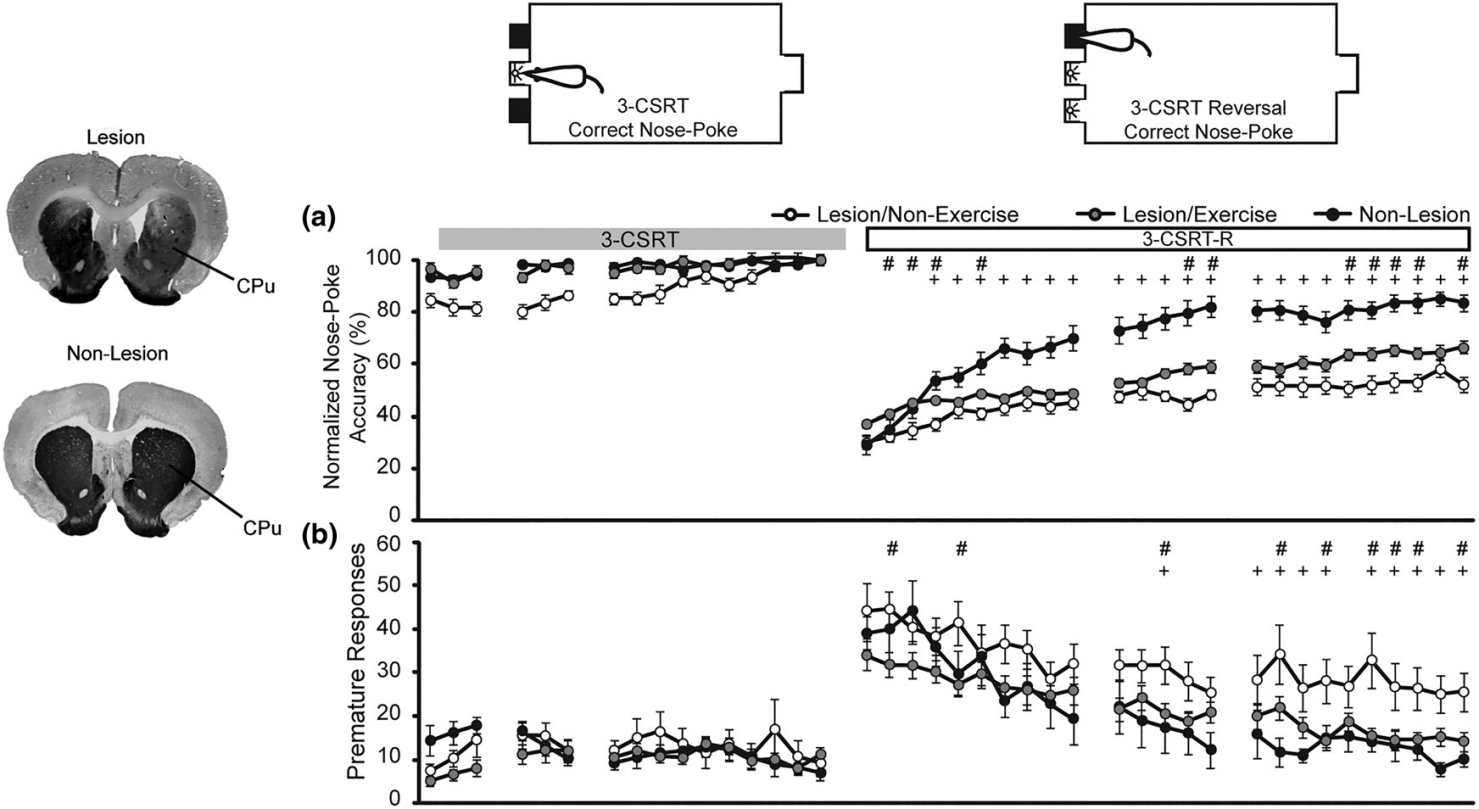
Exercise improves cognitive flexibility in a Parkinsonian rat model. Rats received 6-hydroxydopamine lesions of the bilateral dorsomedial CPu. Animals were exercised for 12 weeks, followed by tyrosine hydroxylase staining of the brain. Lesion/exercise rats compared to Lesion/Non-Exercise rats showed a modest improvement in processing-related responses in a 3-choice serial reaction time (3-CSRT) task and significant improvement during rule reversal of this task (3-CSRT-R). Cognitive flexibility is assessed by improved (a) response accuracy (nose-poke) and (b) inhibitory aptitude (premature responses). Avrg ±SEM, ^#^ *p* < 0.05 lesion/exercise (*n* = 22) vs lesion/sedentary (*n* = 12); ^+^ *p* < 0.05 lesion/sedentary (*n* = 12) vs sham/sedentary (*n* = 12), Fisher’s LSD multiple comparisons test. Figure adapted from Wang et al. [149] with permission from Elsevier.

Our findings in the 6-OHDA PD rodent model demonstrated small but significant exercise-related improvements to deficits in cognitive flexibility (Figure 6) [149]. Exercise improved nose-poke accuracy in lesioned animals during initial learning of a 3-choice serial reaction time (3-CSRT) task. However, lesion/nonexercise rats were able to achieve levels of accuracy equivalent to those of the controls and lesioned/exercised rats by days 14–16 of training – findings suggesting that dopaminergic deafferentation delays memory consolidation. The effect of exercise on cognitive improvement was most apparent during the rule reversal phase (3-CSRT-R). Here, improvements in accuracy in lesion rats undergoing exercise were progressive, with improvement relative to lesion/nonexercise rats maintained after 25 daily exercise sessions. Cognitive gains, though significant, were modest. Results were consistent with those of a recent meta-analysis, with similar findings in healthy human subjects [150] and PD patients [151–153]. A greater effect of exercise in lesioned rats was seen on decreases in premature responses, a measure of behavioral impulsivity. These findings mirror a report in PD patients, where six weeks of intermittent aerobic walking elicited significant improvement in cognitive inhibition (Flanker test) but not on set shifting (Wisconsin card sort, Trail Making tests) [154]. Others have observed improvements in inhibitory aptitude (Stroop test) but not in cognitive flexibility (Trail making test) in PD patients following 3 months of intermittent aerobic cycling [155].

### 4.2 Does the type of exercise matter in PD functional recovery?

Little is known regarding which type of exercise is most beneficial in the neurorehabilitation of neurodegenerative disorders and, more specifically, how to optimize the parameters of exercise (e.g., intensity, duration, complexity, frequency, and volume) to elicit maximal functional recovery. This is relevant as previous work has suggested that different types of physical activity may elicit different physiologic brain responses. Specifically, aerobic exercise has been reported to increase the density of blood capillaries (angiogenesis) and the birth of new neurons (neurogenesis) [156,157]. Conversely, skilled exercise may increase neuronal sprouting and synaptogenesis, which is important for re-establishing brain circuits impaired in disease states and required for normal behaviors, including cognition [158–161]. In fact, studies of normal aging provide evidence that skilled exercise (complex, coordinated, requiring higher processing) and motor fitness facilitate brain function and cognition in older adults, greater than cardiovascular and aerobic exercise, respectively [150,162]. Recent meta-analyses suggest that skilled exercise such as dance [163,164] may be more efficacious than power training, boxing, or resistance exercise [165,166], with ongoing work examining whether different exercise paradigms and parameters of training exert differential effects on specific motor outcomes such as functional mobility, balance, motor function, and gait speed [164,167–169].

It has been suggested that the type of exercise may shape regional brain changes. When comparing skilled to nonskilled exercise, our results in the lesioned animals showed that skilled compared to nonskilled training resulted in greater enhancement of prefrontal cortex- and cerebellar-mediated control of motor function while eliciting equal or greater recovery in behavioral motor deficits. Following 4 weeks of skilled exercise, lesioned animals showed increases in rCBF during walking the prefrontal cortex (prelimbic area), broad areas of the somatosensory cortex, and the cerebellum, whereas those exposed to nonskilled training showed greater activation in the dorsal CPu and dorsal hippocampus [170].

### 4.3 Focus on strengthening frontal cortical inputs

Neural adaptation in the prefrontal cortex may depend in part on the stage of disease. Early in the disease course, patients may be able to recruit the prefrontal cortex during a dual-task walk, whereas, in more advanced diseases, this ability diminishes [171,172]. It has been suggested that motor rehabilitation programs for PD patients should include a relatively high cognitive demand, such that by engaging patients to practice task-switching, they might be able to overcome their exaggerated context dependency [173,174]. Our findings in the rodent model showed that, indeed, when animals walk in a complex wheel with irregular rung spacing – an acute motor challenge requiring ongoing decisions regarding foot placement – they show greater functional connectivity in the medial prefrontal-striatal circuit than when they walk in a smooth running wheel [175]. Based on this observation, we hypothesized that animals performing chronic exercises in the complex wheel would show greater cognitive improvements in the 3-CSRT operant task than animals running either on a simple smooth wheel or those running on a horizontal treadmill. While our study was underpowered to detect subtle differences, cognitive improvements were largely equivalent across groups [149]. This was contrary to our expectations and emphasized the challenge of designing and delivering long-term skilled exercises that remain cognitively engaging. This underscores the fact that learning a skilled task is a dynamic process. Even a simple task such as slow treadmill walking will, at its initiation, require the engagement of frontal systems, attention, and executive function, and thus, initially, may be considered complex. Furthermore, in running mice in groups on a horizontal treadmill, there is much switching of lanes and circumnavigation between animals, making this purportedly “simple” task one that requires ongoing internal guidance. Conversely, complex tasks, once learned, may become habitual, and hence, over time, will require less supervision by the frontal cortex to become increasingly “simple.”

The fact that “the type of exercise matters” is supported by a recent meta-analysis in healthy human subjects, where there appears to be higher cognitive benefits after coordinative exercise compared to endurance, resistance, and mixed exercise [150]. In contrast, a recent systematic review of randomized controlled trials of physical exercise programs on cognitive function in PD reported that exercise-related improvements in global cognitive function, processing speed, sustained attention, and mental flexibility showed the largest effect for intense treadmill exercise, not for skilled exercise (tango or cognitive exercise associated with motor exercise) [134]. This underscores the potential importance of not only skill but also exercise intensity as an important parameter in cognitive neurorehabilitation. The question “What is the best exercise in PD?” may thus be better rephrased as “What is the best exercise for what purpose?” Current research argues for a differential effect of different exercise modalities and parameters on outcome measures of mobility, balance, motor function, gait speed, mood,` and cognition.

### 4.4 Focus on strengthening cerebellar inputs

If indeed recruitment of the cerebellar-thalamocortical circuit is a compensatory strategy elicited by basal ganglia injury, this would suggest that neurorehabilitation, especially early in the disease, might benefit from the incorporation of balance exercises that specifically engage the cerebellum. However, results of randomized controlled trials remain divergent, with both benefits [176–179] and no significant changes reported [180]. Furthermore, while some studies suggested a possible efficacy of cerebellar transcranial magnetic stimulation (TMS) [181–184], other studies have reported no significant improvement [185,186].

A review of clinical studies over the past decade focused on the question of reactive changes in the cerebellum of PD patients shows a complicated picture. Cerebellar hyperactivation may be seen early in the disease [187–189], especially in motor tasks with significant cognitive demands [190,191] and during internally guided limb movements [56]. Also reported has been increased functional cerebellar connectivity reorganization immediately after a single levodopa dose [192,193]. Such cerebellar recruitment may provide some element of compensation [194]. However, cerebellar neuroadaptive changes in response to basal ganglia deficits may also prove to be dysfunctional [195], especially in the later course of the disease. It has been proposed that dopamine controls tremor in PD by inhibiting the cerebellar thalamus (ventral intermediate nucleus) and that this inhibition diminishes when there is the greatest loss of dopaminergic tone [196]. Excessive cerebellar connectivity has been linked to abnormal cerebellar oscillations [197,198], may contribute to dyskinesia [199,200], and has been linked to a “tremor dominant” subtype [201–203], as well as possibly to an “impulsive-compulsive” behavioral subtype [204] and a “freezing of gait” subtype [205]. Thus, recruitment of the Cb-T-C circuit early in the course of PD may represent the brain’s attempt to compensate for early motor deficits. However, later in the course of illness, such adaptive changes may come to represent a vulnerability for developing dyskinesias and tremors. Improved scientific understanding of such adaptive strategies is needed, specifically as it relates to the question of functional versus dysfunctional recruitment.

## 5 Future challenges

### 5.1 Parsing differences between functional compensation, silencing, or maladaptation

#### 5.1.1 Terminology matters

**Figure 7:**
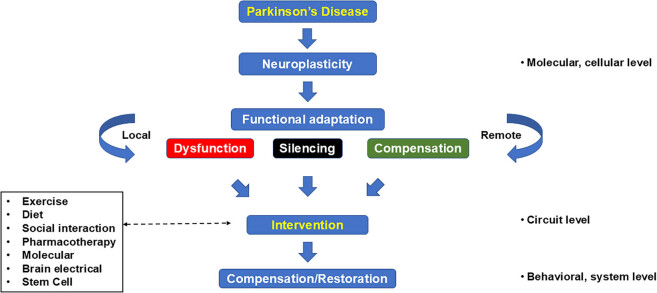
Neuroplastic response: Functional compensation, silencing, or maladaptation in the Parkinsonian brain.

Are changes in rCBF or CMR after local brain injury related to functional *compensatory* mechanisms of the brain? Are they simply the manifestations of a damaged, *silenced* circuit? Or are they *maladaptive* in a manner that impedes functional recovery (Figure 7)? Differentiation between these neuroplastic changes is important in the interpretation of neuroimaging studies. Foremost is the need to properly assess the meaning of changes in the functional brain map of PD patients, with the understanding that cerebral reorganization by itself does not guarantee improvements in functional outcomes. While certain changes may directly relate to dopaminergic deficits, this is only part of the story, and compensatory and/or maladaptive nondopaminergic changes may be seen, both in the classical BG-T-C circuit as well as outside of it. It is important to look at correlations of behavioral improvements related to imaging changes in individual brain regions or subnetworks. Ideally, investigations should include a dose–response relationship between behavioral change and alterations in the functional brain map. Improved understanding here promises to expand our theoretical conceptualization of the disease process, to aid in better defining resilience and prognosis of the PD brain, as well as to provide potential new treatment targets.

In preclinical models, correlations between behavioral improvements and changes in underlying cellular metabolism (e.g., cytochrome oxidase histochemistry and lactate uptake), molecular changes (e.g., receptors and second messengers), and markers of plasticity (e.g., dendritic spines, plasticity markers, and neurotrophins) may provide a scientific foundation to begin to distinguish between compensation, silencing, and maladaptation. In animal models, concomitant brain mapping of changes in the cerebral structure (MRI, CT) and function (glucose uptake and rCBF) will provide an animal-to-human translational bridge to aid in the interpretation of human brain mapping studies that use similar outcome measures.

### 5.2 Limits of preclinical models

Modeling an illness such as PD that is heterogeneous in both etiology and clinical manifestations remains challenging. Unsurprisingly, no single animal model encompasses all of the behavioral, physiologic, and histologic changes. While some animal models parallel motor deficits remarkably well [2,3], others may be more appropriate for studies modeling aspects of alpha-synuclein deposition, inflammation, mitochondrial dysfunction, pesticide-associated pathology, or PD-associated genes and transcription factors (for review, see Dovonou et al. [206]). Matching the optimal animal model to the study question is essential.

### 5.3 Clinical challenges

Both plasticity and degenerative events may co-occur over long periods, not only in the 6-OHDA model but also in human PD. Furthermore, neural recruitment has the potential to elicit functional as well as dysfunctional outcomes. Hence, early intervention may differ from intervention in the late stages of the illness. Whether different types of exercise can preferentially target subregions of the brain to improve motor and cognitive outcomes remains an area of active research, though components are already being integrated into the physical rehabilitation of PD patients [207]. Beyond the question of the type of intervention, its duration, and intensity, a number of questions remain to be addressed: Can an intervention help “generalize” motor learning beyond the confines of the learned task [208,209]? Can combined treatments, such as drug or TMS augmentation of exercise, offer additional benefits [210,211]? Does experience-specific reorganization of the brain aid or impede the acquisition of new behaviors or the performance of old behaviors? Such proactive and retroactive interference of motor behaviors has long been recognized but lacks a sound scientific explanation [50,212–214].

We anticipate that circuit-based approaches to functional brain reorganization will help guide future behavioral, electrical, or molecular strategies to augment targeted neurorehabilitation. Relevant here is that functional disconnections can occur not only in gray matter but also in white matter. In fact, in PD subjects, impaired cross-hemispherical white matter connectivity may occur prior to nigrostriatal dopamine loss and cognitive decline. Later stages of illness show involvement of the corpus callosum when patients demonstrate mild cognitive impairment, leading to dementia after disruption of fronto-parieto-temporal structural connectivity [215,216]. White matter lesions in the “disconnectome” may themselves be potential targets, either for predicting disease progression, for exploring nonmotor symptoms, as markers of neuroplasticity-driven rehabilitation, or as pharmacologic targets (e.g., anti-inflammatory strategies or enhancement of myelination, axon regeneration or energy metabolism).

Molecular, histologic, and neuroimaging studies in animal models will help inform the interpretation of findings from clinical neuroimaging (MRI, fMRI, PET, and SPECT) and are positioned to more easily answer questions of causality and mechanism. Results should be corroborated in clinical research settings using pharmacoimaging, diffusion tensor imaging, and postmortem tissue analysis. Interpretation of results will need to take into account the stage of illness and PD subtypes (e.g., tremor-dominant, posturally unstable/gait impaired, familial, early onset versus late onset, and nonmotor versus motor-dominant), as well as whether patients are on or off l-dopa at the time of imaging [217]. Important in this translational approach is to also consider the question of sex differences which in PD patients have been reported in the extent of cortical and subcortical atrophy [218,219], mesolimbic functional connectivity [220], functional efficiency of white matter connectivity [218], brain energy metabolism [221], striatal [^18^F]-fluoro-l-dopa uptake [222], neurophysiologic brain response to transcranial magnetic stimulation [223], and cognitive improvements after exercise [150]. Graph theory has substantially contributed to our understanding of networks of the brain and has been proposed as a theoretical way to explain the observations of functional reorganization of the brain following injury. However, to date, the predictive value of graph theoretical measures of brain function has not been extensively tested in PD.
